# Association of Age With Likelihood of Developing Symptoms and Critical Disease Among Close Contacts Exposed to Patients With Confirmed SARS-CoV-2 Infection in Italy

**DOI:** 10.1001/jamanetworkopen.2021.1085

**Published:** 2021-03-10

**Authors:** Piero Poletti, Marcello Tirani, Danilo Cereda, Filippo Trentini, Giorgio Guzzetta, Giuliana Sabatino, Valentina Marziano, Ambra Castrofino, Francesca Grosso, Gabriele Del Castillo, Raffaella Piccarreta, Aida Andreassi, Alessia Melegaro, Maria Gramegna, Marco Ajelli, Stefano Merler

**Affiliations:** 1 Fondazione Bruno Kessler, Povo, Trento, Italy; 2 Directorate General for Health, Lombardy Region, Milan, Italy; 3 Health Protection Agency of Pavia, Pavia, Italy; 4 Department of Biomedical Sciences for Health, University of Milan, Milan, Italy; 5 Dondena Centre for Research on Social Dynamics and Public Policy, Bocconi University, Milan, Italy; 6 Department of Decision Sciences, Bocconi University, Milan, Italy; 7 CovidCrisisLab, Bocconi University, Milan, Italy; 8 Department of Social and Political Sciences, Bocconi University, Milan, Italy; 9 Department of Epidemiology and Biostatistics, Indiana University School of Public Health, Bloomington; 10 Laboratory for the Modeling of Biological and Socio-technical Systems, Northeastern University, Boston, Massachusetts

## Abstract

**Importance:**

Solid estimates of the risk of developing symptoms and of progressing to critical disease in individuals infected with severe acute respiratory syndrome coronavirus 2 (SARS-CoV-2) are key to interpreting coronavirus disease 2019 (COVID-19) dynamics, identifying the settings and the segments of the population where transmission is more likely to remain undetected, and defining effective control strategies.

**Objective:**

To estimate the association of age with the likelihood of developing symptoms and the association of age with the likelihood of progressing to critical illness after SARS-CoV-2 infection.

**Design, Setting, and Participants:**

This cohort study analyzed quarantined case contacts, identified between February 20 and April 16, 2020, in the Lombardy region of Italy. Contacts were monitored daily for symptoms and tested for SARS-CoV-2 infection, by either real-time reverse transcriptase–polymerase chain reaction using nasopharyngeal swabs or retrospectively via IgG serological assays. Close contacts of individuals with laboratory-confirmed COVID-19 were selected as those belonging to clusters (ie, groups of contacts associated with an index case) where all individuals were followed up for symptoms and tested for SARS-CoV-2 infection. Data were analyzed from February to June 2020.

**Exposure:**

Close contact with individuals with confirmed COVID-19 cases as identified by contact tracing operations.

**Main Outcomes and Measures:**

Age-specific estimates of the risk of developing respiratory symptoms or fever greater than or equal to 37.5 °C and of experiencing critical disease (defined as requiring intensive care or resulting in death) in SARS-CoV-2–infected case contacts.

**Results:**

In total, 5484 case contacts (median [interquartile range] age, 50 [30-61] years; 3086 female contacts [56.3%]) were analyzed, 2824 of whom (51.5%) tested positive for SARS-CoV-2 (median [interquartile range] age, 53 [34-64] years; 1604 female contacts [56.8%]). The proportion of infected persons who developed symptoms ranged from 18.1% (95% CI, 13.9%-22.9%) among participants younger than 20 years to 64.6% (95% CI, 56.6%-72.0%) for those aged 80 years or older. Most infected contacts (1948 of 2824 individuals [69.0%]) did not develop respiratory symptoms or fever greater than or equal to 37.5 °C. Only 26.1% (95% CI, 24.1%-28.2%) of infected individuals younger than 60 years developed respiratory symptoms or fever greater than or equal to 37.5 °C; among infected participants older than 60 years, 6.6% (95% CI, 5.1%-8.3%) developed critical disease. Female patients were 52.7% (95% CI, 24.4%-70.7%) less likely than male patients to develop critical disease after SARS-CoV-2 infection.

**Conclusions and Relevance:**

In this Italian cohort study of close contacts of patients with confirmed SARS-CoV-2 infection, more than one-half of individuals tested positive for the virus. However, most infected individuals did not develop respiratory symptoms or fever. The low proportion of children and young adults who developed symptoms highlights the possible challenges in readily identifying SARS-CoV-2 infections.

## Key Points

**Question:**

What is the association of age with the likelihood of developing respiratory symptoms or fever greater than or equal to 37.5 °C and the association of age with the likelihood of progressing to critical disease after severe acute respiratory syndrome coronavirus 2 (SARS-CoV-2) infection?

**Findings:**

In this cohort study, 5484 quarantined case contacts were monitored daily for symptoms for at least 2 weeks and were tested for infection via real-time reverse transcriptase–polymerase chain reaction or serological screening more than 1 month after identification. Only 26.1% of infected individuals younger than 60 years developed respiratory symptoms or fever greater than or equal to 37.5 °C, but 6.6% of infected participants aged 60 years or older developed critical disease.

**Meaning:**

The low proportion of children and young adults who developed symptoms highlights the possible challenges in readily identifying SARS-CoV-2 infections.

## Teaser

This cohort study examines the association of age with the likelihood of experiencing symptoms and progressing to critical illness after infection with severe acute respiratory syndrome coronavirus 2 (SARS-CoV-2) in Italy.

## Introduction

The role of asymptomatic infections in the transmission of severe acute respiratory syndrome coronavirus 2 (SARS-CoV-2) is still an important missing piece in the puzzle of the ongoing pandemic.^1,2,3,4,5^ The second coronavirus disease 2019 (COVID-19) epidemic wave experienced in many industrialized countries^6^ has highlighted the challenges of containing COVID-19 outbreaks via contact tracing strategies alone, the effectiveness of which is limited by the ability of asymptomatic carriers to spread infection.^7,8,9,10^ Quantifying the age-specific proportion of SARS-CoV-2–infected individuals who do not develop recognizable symptoms is, thus, key to understanding COVID-19 epidemic trajectories and identifying the settings and age segments of the population in which silent transmission is more likely to occur.^2,5,6,7,8,9,10^ Robust estimates of the age-specific risk of requiring critical care upon infection are also needed to assess and project the health care burden of COVID-19 and to guide the allocation of resources when planning for new surges of cases.^11,12,13^

In this cohort study, we retrospectively analyzed clinical observations of quarantined close contacts of confirmed cases identified during surveillance activities conducted in Lombardy, Italy, between February and April 2020. The analyzed sample was obtained by combining different sources of information on the same set of individuals and applying strict exclusion criteria. The resulting set of study participants was previously analyzed to estimate age-specific infection fatality ratios.^14^ In this study, instead of infection fatality ratios, we leveraged the continuous monitoring of clinical manifestations and outcomes among case contacts to investigate the association between SARS-CoV-2 infection and development of symptoms and/or critical disease. In particular, we investigated whether a participant developed (1) respiratory symptoms or fever of 37.5 °C or higher or (2) critical disease, defined as disease requiring admission to an intensive care unit or disease leading to death with a diagnosis of SARS-CoV-2 infection. The aim of this study is to identify the association of age with the likelihood of developing symptoms and the association of age with the likelihood of progressing to critical illness after SARS-CoV-2 infection.

## Methods

### Study Population

Data collection and analysis were part of outbreak investigations conducted during a public health emergency. The processing of COVID-19 data is necessary for reasons of public interest in the area of public health, such as protecting against serious cross-border threats to health or ensuring high standards of quality and safety of health care; therefore, this study was exempted from institutional review board approval and the need for informed consent (Regulation EU 2016/679 GDPR). This study followed the Strengthening the Reporting of Observational Studies in Epidemiology (STROBE) reporting guideline.

Lombardy was the earliest and most affected region in Italy during the first wave of the COVID-19 epidemic. Shortly after the detection of the first locally acquired infection on February 20, 2020, geographically targeted interventions were immediately implemented, such as the ban on mass gatherings and the definition of quarantined zones around the most affected municipalities. In quarantined zones, nonessential productive activities were suspended, and strict individual movement restrictions were applied. On March 8, 2020, strict measures were imposed for the whole region and extended shortly after to the rest of country, banning nonessential travel and public events, limiting movement except in cases of necessity, closing commercial and retail businesses except essential goods sellers and banks, suspending teaching in schools and universities, and shutting down all unnecessary businesses and industries. Suspended economic and social activities were gradually resumed between April 14 and May 18, 2020.

We analyzed close contacts of COVID-19 cases identified in Lombardy during contact tracing activities conducted by local health authorities, between February 21 and April 16, 2020. Cases were determined by using real-time reverse transcription–polymerase chain reaction (RT-PCR) testing on nasopharyngeal swabs.^15,16,17^ RT-PCR testing was performed by both private and public laboratories that are part of the national health service, which is organized under the Ministry of Health and is administered on a regional basis. Infections ascertained by the surveillance system were considered as potential index cases of a cluster, although they were not necessarily the first infector of their cluster. Close contacts of laboratory-confirmed cases were identified through standardized epidemiological investigations to determine the history of individuals’ exposure. For individuals who were unable to participate in the contact tracing interview, close contacts were identified by their emergency contacts. Close contacts of cases were defined as persons living in the same household of a case or who engaged face-to-face with a case patient within a distance of 2 m for more than 15 minutes during the exposure period. The exposure period was initially defined as the interval ranging from 14 days before to 14 days after the date of symptom onset of the index case of the cluster (ie, groups of contacts identified by 1 positive index case). After March 20, the period was shortened, ranging from 2 days before to 14 days after the symptom onset of the index case.^18^

Close contacts of cases were identified and informed of their possible exposure within 24 to 48 hours after a positive test result for the index case. All identified contacts were quarantined, followed up daily for at least 14 days,^15,19^ and required by Italian regulations to report symptoms to public health authorities. From February 20 to February 25, 2020, all contacts of confirmed COVID-19 cases were tested with RT-PCR, irrespective of clinical symptoms. From February 26 onward, the traced contacts were tested only in case of symptom onset. Positive individuals were isolated, and their data were recorded in a line list of laboratory-confirmed infections where their clinical outcome was regularly updated. On April 16, 2020, Lombardy initiated a serological survey aimed at detecting IgG neutralizing antibodies against S1 and S2 antigens of SARS-CoV-2 in contacts of the identified index cases.^20,21^ Serological testing was performed using automated Liaison SARS-CoV-2 S1 and S2 IgG assay (94.4% sensitivity at 15 days from diagnosis; specificity, 98.3%).^20^ Serological test results were binary and communicated to tested participants, who were categorized as seropositive if they had developed IgG antibodies.

Identification of close contacts and RT-PCR testing were conducted between February 21 and April 27, 2020. This range refers to dates of laboratory diagnosis for contacts and index cases. Screening for IgG antibodies against SARS-CoV-2 was performed between April 16 and June 15, 2020. The follow-up for clinical outcomes, including possible patients’ admission to intensive care units or death, was conducted until June 8, 2020. Data collection, storage, anonymization, and management were performed by regional health authorities as part of surveillance activities, and outbreak investigations were conducted to mitigate the COVID-19 epidemic (the process of obtaining surveillance data is described in more detail in eAppendix 1 in the Supplement).

### Sample Description

Study participants were selected from an initial database of 62 881 close contacts of 21 519 confirmed SARS-CoV-2 infections (index cases). To minimize potential biases associated with the identification of infections, we selected a sample consisting of contacts belonging to clusters whose individuals were all tested for SARS-CoV-2 infection, either through RT-PCR testing of nasal swabs during the contact tracing operations or through retrospective IgG serological testing performed at least 1 month after exposure. Contacts identified after April 16, 2020, were excluded to avoid biases caused by delays in symptom development, seroconversion of infected individuals, or potential exposure after the lifting of restrictions imposed during the national lockdown. More specifically, 90 clusters (0.42%) were excluded by the proposed analysis because of 101 contacts (0.16% of all case contacts) with inconclusive serological results; 18 007 clusters (83.7%) were excluded because of incomplete testing. Participants identified as contacts by more than 1 positive case were considered only once; 5 case contacts were excluded because of incomplete information on age. Although they were not necessarily the first infections in the identified clusters, index cases were excluded from our analysis because they were often identified by their symptoms and may, therefore, have been at higher risk of experiencing symptoms or severe disease.

Participants were defined as positive for SARS CoV-2 infection if they had at least 1 laboratory confirmation (either via RT-PCR or serological assay), regardless of clinical signs. Infected participants were defined as symptomatic if they had upper or lower respiratory tract symptoms or fever of 37.5 °C or higher. Respiratory symptoms included dry cough, dyspnea, tachypnea, difficulty breathing, shortness of breath, sore throat, and chest pain or pressure. This list of respiratory symptoms was used consistently by local health authorities throughout the entire study period. Critical cases were defined as patients either admitted to an intensive care unit or deceased with a diagnosis of SARS-CoV-2 infection.

### Statistical Analysis

The primary outcomes of our analysis are the age-specific association with the likelihood of developing symptoms after infection and the age-specific association with the likelihood of progressing to critical disease after infection. Proportions stratified by age and sex are provided as the crude ratio between the number of symptomatic infected individuals (or critical patients) and the total number of infected individuals. The relative risk (RR) of developing symptoms and critical disease in 5 discrete 20-year age groups was estimated by using a generalized linear mixed-effects model with logit link and cluster-specific random effects. The model included as covariates the individual’s age group and sex and the number of symptomatic contacts in the cluster. The model was selected through a stepwise procedure to rule out alternative models on the basis of likelihood ratio tests. More details are reported in eAppendix 2 in the Supplement. RRs were computed with conditioning on the covariates. Differences in the RRs across multiple groups were assessed by using 1-way analysis of variance, followed by post hoc Tukey test. Potential biases introduced by selecting only clusters with complete testing were considered by exploring whether contacts of milder index cases got tested less frequently. Specifically, a 2-sided *t* test was used to assess whether the proportion of tested contacts was significantly different between contacts of symptomatic and asymptomatic index cases. Significance was defined as *P* < .05. Statistical analysis was performed using R statistical software version 3.6.2 (R Project for Statistical Computing). Data were analyzed from February to June 2020.

In our baseline analysis, contacts identified by more than 1 positive case were associated with the cluster of the first identified positive index case. The effect of alternative grouping was explored by replicating our analysis for 1000 data sets, where contacts with multiple index cases were randomly assigned to 1 of the clusters to which they belonged. Estimates were compared in terms of mean and 95% CI of estimated RRs of developing symptoms for different age groups.

To explore to what extent false-negative RT-PCR results could have affected our estimates, we computed the proportion of symptomatic infections resulting from 10 000 simulations where a random sample of contacts who were RT-PCR–negative contacts and were not serologically tested was assumed to be SARS-CoV-2 positive. Similarly, to explore to what extent false positives arising from IgG testing (98.3% specificity) may have impacted our estimates, we repeated our analysis using 10 000 simulations where a random sample of 1.7% contacts positive for IgG that were not confirmed by RT-PCR results were assumed to be SARS-CoV-2 negative.

## Results

In the overall data set (62 881 close contacts), a similar percentage of tested individuals was found for clusters with symptomatic and asymptomatic index cases (23.6% vs 25.2%). We selected 3420 clusters where all contacts were tested against SARS-CoV-2 infection either via nasal swabs during follow-up of contact tracing activities or within the serological survey (eFigure 1 in the Supplement). The analyzed sample thus consisted of 5484 close contacts (median [interquartile range] age, 50 [30-61] years; 3086 female contacts [56.3%]); index cases were excluded. Complete information was available for all of these contacts. Among the selected sample, 2824 (51.5%) had been infected (median [interquartile range] age, 53 [34-64] years; 1604 female contacts [56.8%]). Of the 2824 confirmed SARS-CoV-2 infections, 876 patients (31.0%) developed respiratory symptoms or fever of 37.5 °C or higher; 75 cases (2.7%) were critical. Most infected contacts (1948 of 2824 individuals [69.0%]) did not develop respiratory symptoms or fever of 37.5 °C or higher.

Data stratified by sex and age are displayed in the Table. The likelihood of developing symptoms upon infection increased with age, ranging from 18.1% (95% CI, 13.9%-22.9%) among participants younger than 20 years to 64.6% (95% CI, 56.6%-72.0%) for participants aged 80 years or older (Figure). Overall, the likelihood of developing these symptoms in participants younger than 60 years was 26.1% (95% CI, 24.1%-28.2%); only 0.54% (95% CI, 0.26%-1.00%) of participants younger than 60 years developed critical disease, in striking contrast to 6.6% (95% CI, 5.1%-8.3%) among participants aged 60 years or older. Female participants had a lower likelihood of developing critical disease compared with male participants (2.06% [95% CI, 1.42%-2.88%] vs 3.44% [95% CI, 2.49%-4.63%]).

**Table.** Sample Description and Estimates by Sex and Age Group

Characteristic	Participants, No.		Patients, No. (%) [95% CI]	
	Total	Positive for SARS-CoV-2 infection^a^	Symptomatic infections^b^	Critical illness
Sex				
Male	2398	1220	371 (30.41) [27.84-33.08]	42 (3.44) [2.49-4.63]
Female	3086	1604	505 (31.48) [29.22-33.82]	33 (2.06) [1.42-2.88]
Age group, y				
0-19	692	304	55 (18.09) [13.93-22.89]	0 (0) [0-1.21]
20-39	1177	531	119 (22.41) [18.93-26.2]	2 (0.38) [0.05-1.35]
40-59	2015	1002	306 (30.54) [27.7-33.49]	8 (0.8) [0.35-1.57]
60-79	1352	829	294 (35.46) [32.2-38.83]	36 (4.34) [3.06-5.96]
≥80	248	158	102 (64.56) [56.56-71.99]	29 (18.35) [12.65-25.28]
Total	5484	2824	876 (31.02) [29.32-32.76]	75 (2.66) [2.09-3.32]

Abbreviation: SARS-CoV-2, severe acute respiratory syndrome coronavirus 2.

^a^ Participants were defined as positive for SARS-CoV-2 infection if they had at least 1 laboratory confirmation (either via reverse transcriptase–polymerase chain reaction or serological assay), irrespective of clinical signs or symptoms.

^b^ Symptomatic infection was defined as the presence of respiratory symptoms or fever greater than or equal to 37.5 °C.

**Figure.**
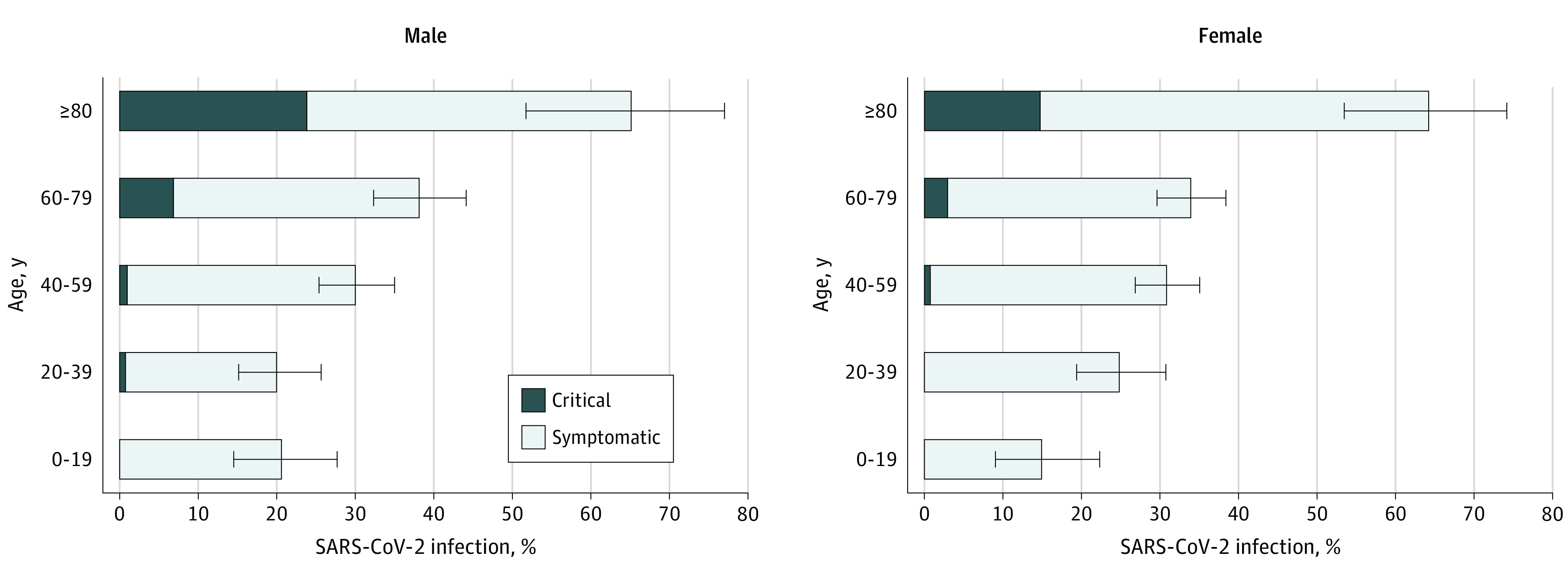
Estimated Percentage of Symptomatic Severe Acute Respiratory Syndrome Coronavirus 2 (SARS-CoV-2) Infections and Critical Disease in Male and Female Contacts Across Different Age Groups Symptomatic SARS-CoV-2 infection was defined as having respiratory symptoms or fever of 37.5 °C or higher. Critical disease was defined as requiring intensive care or resulting in death. Horizontal lines represent 95% CIs computed by exact binomial tests.

By considering a set of possible confounding factors and cluster effects, the modeling analysis supports the association of age with both the risk of developing symptomatic infection and the risk of progressing to critical illness after infection found in the baseline analysis. In particular, SARS-CoV-2–positive participants older than 60 years had a higher risk of developing symptoms compared with all younger age groups, whereas infections in participants younger than 20 years resulted in a lower risk of developing symptoms compared with older participants (eFigure 2 in the Supplement). No significant differences between SARS-CoV-2–infected female and male patients were found in the risk of developing symptoms, whereas female patients were 52.7% (95% CI, 24.4-70.7) less likely to experience critical disease than male patients. These results were obtained by associating contacts with multiple index cases to the first identified positive index case. Similar results were obtained when these contacts were randomly assigned to any of their index cases (eFigure 2 in the Supplement).

In our sample, 137 of 327 RT-PCR–negative participants (41.9%) tested both by RT-PCR and serology were IgG positive (eTable in the Supplement). To assess the robustness of our estimates with respect to failures in RT-PCR testing, we considered the worst-case scenario where 41.9% of 732 contacts who were tested only via RT-PCR and had negative results were assumed to be false-negative contacts. In this case, the estimated fraction of symptomatic infections was 37.5% (95% CI, 35.8-39.2) vs 31.0% (95% CI, 29.3-32.8) estimated in the main analysis. Similarly, to explore how possible IgG false-positive results could have affected our findings, we considered a worst-case scenario assuming that 1.7% (32 participants) of the 1892 IgG-positive contacts without a positive RT-PCR result were actually false positive (eTable in the Supplement). In this case, 32.5% (95% CI, 30.8-34.3) of infections were symptomatic.

## Discussion

The contribution of asymptomatic carriers to transmission of SARS-CoV-2 is still poorly quantified,^1,2,4,15,22^ but episodes of transmission from symptom-free, positive individuals have been widely documented.^2,3,7^ Because asymptomatic infections are easily missed by surveillance systems, they can reduce the effectiveness of the test, trace, and isolate strategies in keeping SARS-CoV-2 transmission under control.^23,24^ Our findings may be of particular relevance to understanding SARS-CoV-2 transmission patterns in schools.^8,9^ In fact, we estimated that children have a low risk of developing fever or respiratory symptoms, but according to recent evidence,^9,10^ they are capable of transmitting infection, thus possibly becoming a source of silent SARS-CoV-2 transmission.

Aggregated estimates of the proportion of SARS-CoV-2 symptomatic infections published so far are highly variable, ranging from 17% to 87%,^1,2,4,22,25^ and they depend on which symptoms are included in the definition, the methods for ascertaining infections, and when infection was ascertained relative to symptom onset. Our estimate of the aggregated risk of developing respiratory symptoms or fever of 37.5 °C or higher (31.0%) is in line with the published medical literature.^2,4^ However, in this study, we provide the association of age with the likelihood of developing these symptoms in SARS-CoV-2–infected individuals. Obtained estimates will be key to identifying social contexts and age segments of the population in which silent transmission of SARS-CoV-2 is more likely to occur. Moreover, the association of age with the risk of developing critical disease after infection provided in this study can be instrumental to informing resource planning, such as required hospital beds and intensive care units, to ensure the sustainability of health care systems.

### Strengths and Limitations

One of the strengths of this study relies on the analyzed sample, consisting of a cohort of close contacts of confirmed index cases who were followed up daily for symptoms. Those participants were either tested via RT-PCR or subsequently examined for previous SARS-CoV-2 infection via serological testing. The clinical outcomes of all participants, including possible admission to intensive care units or death, were regularly updated by the regional health surveillance. This selection procedure allowed us to (1) identify most SARS-CoV-2 infections among the participants (ie, close contacts of confirmed index cases) and (2) markedly reduce the possibility of recall bias in the definition of symptoms for individuals later identified as positive by serological assays. Moreover, the age-specific infection fatality ratio estimated from the same set of participants analyzed here^14^ compares well with that reported by Verity et al.^26^ This provides an indirect validation of the adopted approach in defining a reliable denominator to assess the likelihood of developing symptoms and of progressing to critical disease in infected individuals of different ages.

It is important to emphasize that the sensitivity and specificity of the assays used to confirm infection^16,17,20,21,27^ should be considered when interpreting the results of this analysis. In our sample, RT-PCR testing was conducted during contact tracing; serum samples were collected at least 1 month after identification. Although IgG false negatives due to delays in seroconversion are likely negligible, temporal waning of IgG antibodies among asymptomatic infections cannot be excluded.^28^ However, the 2 sensitivity analyses we conducted confirmed that IgG false-positive and RT-PCR false-negative results would have only slightly biased our estimates of the fraction of symptomatic infections.

It is also important to remark that the infection rate observed in our sample (ie, close contacts of COVID-19 cases) is not representative of that in the general population, because contacts of COVID-19 cases were exposed to a higher risk of infection than the general population, nor does it identify all SARS-CoV-2 infections. Our analysis focuses on a cohort of positive contacts of confirmed index cases who were followed up daily for symptoms and clinical outcomes by health authorities as per Italian regulation. Therefore, the likelihood of developing respiratory symptoms or fever and the likelihood of progressing to critical condition after infection, which represent the primary outcomes of this study, are not affected by the aforementioned limitations. Furthermore, our understanding of symptoms caused by COVID-19 has changed over the course of the pandemic and, although the definition of symptomatic infections has not changed throughout our study, our estimates should be cautiously used to interpret data from geographical areas where different definitions may have been adopted.

## Conclusions

In this cohort study of Italian close contacts of patients with confirmed SARS-CoV-2 infection, most SARS-CoV-2–infected individuals did not develop respiratory symptoms or fever of 37.5 °C or higher, highlighting the challenges of controlling COVID-19 outbreaks with individual-level interventions.

## Notes

Supplement. **eAppendix 1.** Surveillance Data **eFigure 1.** Flowchart Detailing the Inclusions and Exclusions in Our Sample **eTable.** Description of the Sample **eAppendix 2.** Statistical Analysis **eFigure 2.** Results of the Generalized Linear Mixed-Effects Model for the Development of Symptoms After SARS-CoV-2 Infection **eReferences**
