# Supplementary material for: Association of Age With Likelihood of Developing Symptoms and Critical Disease Among Close Contacts Exposed to Patients With Confirmed SARS-CoV-2 Infection in Italy
Source: JAMA Netw Open. 2021 Mar 10;4(3):e211085. doi: 10.1001/jamanetworkopen.2021.1085 (PMC7948061; doi:10.1001/jamanetworkopen.2021.1085)
Supplement: Supplement. — eAppendix 1. Surveillance Data eFigure 1. Flowchart Detailing the Inclusions and Exclusions in Our Sample eTable. Description of the Sample eAppendix 2. Statistical Analysis eFigure 2. Results of the Generalized Linear Mixed-Effects Model for the Development of Symptoms After SARS-CoV-2 Infection eReferences [file jamanetwopen-e211085-s001.pdf]

Supplemental Online Content

Poletti P, Tirani M, Cereda D, et al; ATS Lombardy COVID-19 Task Force. Association of age with likelihood of developing symptoms and critical disease among close contacts exposed to patients with confirmed SARS-CoV-2 infection in Italy. *JAMA Netw Open*. 2021;4(3):e211085.
doi:10.1001/jamanetworkopen.2021.1085

eAppendix 1. Surveillance Data
eFigure 1. Flowchart Detailing the Inclusions and Exclusions in Our Sample
eTable. Description of the Sample
eAppendix 2. Statistical Analysis
eFigure 2. Results of the Generalized Linear Mixed-Effects Model for the Development of Symptoms After SARS-CoV-2 Infection
eReferences

This supplemental material has been provided by the authors to give readers additional information about their work.

© 2021 Poletti P et al. *JAMA Network Open.*

eAppendix 1. Surveillance Data

Soon after the detection of first locally acquired infection in the Lombardy region of Italy on February 20, 2020, national and regional health authorities introduced coordinated interventions to control the epidemic spread. Local health authorities of Lombardy set up a surveillance system to detect local transmission of SARS-CoV-2 by extensive testing, to isolate COVID-19 cases, and to identify and quarantine contacts of positive subjects. Quarantine subjects were daily followed-up for symptoms during their entire quarantine period. Clinical conditions of confirmed cases were regularly updated. On April 16, the Lombardy Region initiated a large-scale serological screening of subjects quarantined for COVID-19 to evaluate the prevalence of the infection in this group. The data analyzed in this paper were obtained by building a database that combines the information collected during the contact tracing activities conducted between February and April 2020 by the Lombardy healthcare agencies, with the Lombardy line list of all COVID-19 patients and the test results obtained through the serological survey conducted in the region between April 16 and June 15, 2020. The database provides, among other details, information on the gender and the age of cases and case contacts, the results of PCR tests and serological tests (if any), the outcome of patients, and whether they were admitted to an intensive care unit. Data were collected as part of surveillance activities to control SARS-CoV-2 transmission, so that information on RT-PCR and serological test results consists only of categorical data (i.e., positive, negative, or inconclusive). Serum samples were mainly collected from subjects who were not tested with RT-PCR or from RT-PCR negative subjects.

The identification and monitoring of close case contacts was performed by regional healthcare agencies, through standardized epidemiological investigations of positive cases (or of their relatives) to determine the history of individuals' exposure. The exposure period was initially defined as the interval time ranging from 14 days before to 14 days after the symptom onset of the index case. After March 20, the time period was reduced to from 2 days before to 14 days after the symptom onset of the index case [1].

According to criteria initially defined by the European Centre for Disease Prevention and Control (ECDC), from February 21 to February 25, suspected COVID-19 cases were identified as:

1. patients with acute respiratory tract infection OR sudden onset of at least one of the following: cough, fever, shortness of breath AND with no other aetiology that fully explains the clinical presentation AND at least one of these other conditions: a history of travel to or residence in China, OR patient is a health care worker who has been working in an environment where severe acute respiratory infections of unknown etiology are being cared for;
2. OR patients with any acute respiratory illness AND at least one of these other conditions: having been in close contact with a confirmed or probable COVID-19 case in the last 14 days prior to onset of symptoms, OR having visited or worked in a live animal market in Wuhan, Hubei Province, China in the last 14 days prior to onset of symptoms, OR having worked or attended a health care facility in the last 14 days prior to onset of symptoms where patients with hospital- associated COVID-19 have been reported.

Probable cases were defined as suspect cases for whom testing for virus causing COVID-19 was positive with a specific real-time RT PCR assay detecting the SARS-CoV-2 virus responsible for the COVID-19 (according to the test results reported by the laboratory) OR for whom testing was inconclusive. At any time, confirmed cases were defined as persons with laboratory confirmation of virus causing of SARS-CoV-2 infection, irrespective of clinical signs and symptoms. The Lombardy line list of patients used to build the database analyzed here consists in all confirmed SARS-CoV-2 infections ascertained until June 8, 2020.

A close case contact was defined as a person living in the same household as a COVID-19 confirmed case, a person having had face-to-face interaction with a COVID-19 confirmed case within 2 meters and for more than 15 minutes; a person who was in a closed environment (e.g. classroom, meeting room, hospital waiting room) with a COVID-19 confirmed case at a distance of less than 2 meters for more than 15 minutes; a healthcare worker or other person providing direct care for a COVID-19 confirmed case, or laboratory workers handling specimens from a COVID-19 confirmed case without recommended personal protective equipment (PPE) or with a possible breach of PPE; a contact in an aircraft sitting within two seats (in any direction) of a COVID-19 confirmed case, travel companions or persons providing care, and crew members serving in the section of the aircraft where the index case was seated (passengers seated in the entire section or all passengers on the aircraft were considered close contacts of a confirmed case when severity of symptoms or movement of the case indicate more extensive exposure). Close case contacts were initially considered as contacts occurred

© 2021 Poletti P et al. *JAMA Network Open.*

between 14 days before and 14 days after the onset of symptoms in the case under consideration. After March 20, close case contacts were defined as contacts occurred between 2 days before and 14 days after the case symptom onset [1]. Clusters of contacts were defined as the set of contacts identified by the contact tracing triggered by a positive index case. Contact data used to build the analyzed database consist of records collected between February 21 and April 16, 2020. Contact data collected after April 16, 2020 were excluded to avoid biases caused by reporting delays, delays between exposure and symptom onset, in seroconversion of infected individuals or potential exposure to multiple sources of infection after the lifting of restrictions imposed during the national lockdown.

In accordance with the WHO recommendations, nasal swabs (UTM viral transport ®, Copan Italia S.p.a) from all suspected cases were tested with at least two real-time RT PCR assays targeting different genes (E and RdRp) of SARS-CoV-2 [2]. In addition, a novel quantitative real-time RT PCR targeting an additional SARS-CoV-2 gene (M) was developed (details provided upon request). From February 21 to February 25, all suspected cases and asymptomatic contacts were tested. From February 26 onward, testing was applied only to symptomatic cases and symptomatic case contacts. From March 20, positivity to the nasal swab was also granted for tests that sought a single gene. Inconclusive swabs were repeated to reach the diagnosis.

Serological screening of subjects quarantined for COVID-19 included both symptomatic and asymptomatic case contacts identified through an epidemiological investigation without history of a swab for SARS-CoV-2. The test used to detect SARS-CoV-2 IgG antibodies is the LIAISON® SARS-CoV-2 test (DiaSorin). The LIAISON® SARS-CoV-2 test employs magnetic beads coated with S1 & S2 antigens [3,4]. The antigens used in the tests are expressed in human cells to achieve proper folding, oligomer formation, and glycosylation, providing material similar to the native spikes. This strategy ensures that the antigen-antibody complex forms with the required specificity. The S1 and S2 proteins are both targets to neutralizing antibodies. The test provides the detection of IgG antibodies against S1/S2 antigens of SARS-CoV-2 and the detection of neutralizing antibodies with 97.8% negative agreement and 94.4% positive agreement to Plaque Reduction Neutralization Test (PRNT). The comparison to PRNT was evaluated by testing 304 samples collected during the outbreak from subjects whose PRNT result was available. Performance analyses validating the accuracy of IgG serological tests used can be found in [3,4]. A negative result (<12 AU/mL) indicates the absence or a very low level of IgG antibodies directed against the virus, this occurs in the absence of infection or during the incubation period or in the early stages of the disease. An inconclusive result (12-15 AU/mL) can be interpreted as both a false positive or a false negative and suggests repeating the exam after a week. A positive result (>15 AU/mL) indicates the presence of IgG antibodies and must be interpreted in association with the clinical outcomes and the possible search for the viral genome on the nasopharyngeal swab. The performed analysis is based on all serological test results obtained within June 15, 2020 from serum samples collected before May 24, 2020.

Sample description

In the available data, only a fraction of case contacts has been tested for IgG antibodies, whereas the majority of symptomatic infections had already been confirmed by RT-PCR. As such, to avoid a biased sample where all symptomatic individuals are included, while only a fraction of asymptomatic infections are considered, we analyzed only those clusters where all contacts were tested (either via RT-PCR or serological assay). Specifically, out of 21,519 clusters identified in Lombardy between between February 21 and April 16, 2020, 90 clusters with case contacts with inconclusive results for both PCR and IgG tests were excluded from the proposed analysis; 18,007 clusters were excluded due to incomplete testing of close contacts; 5 case contacts (2 clusters) were excluded because of incomplete information on age. A flow-chart detailing the exclusion criteria adopted to define our sample is shown in eFigure 1; eTable 1 reports summary statistics of the sample analyzed in this study.

© 2021 Poletti P et al. *JAMA Network Open.*

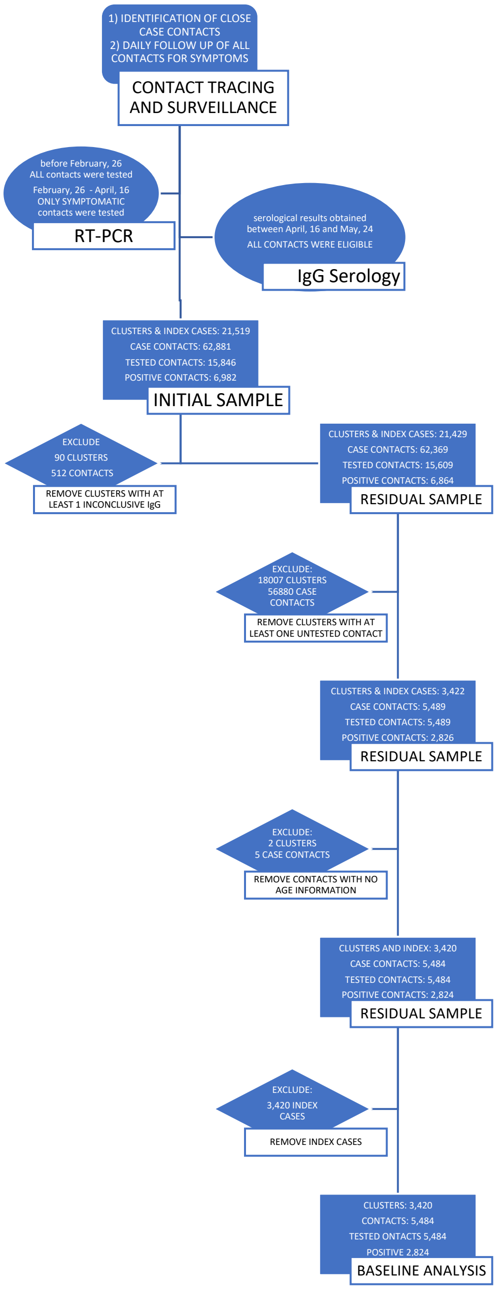

eFigure 1. Flowchart Detailing the Inclusions and Exclusions in Our Sample

© 2021 Poletti P et al. *JAMA Network Open.*

eTable. Description of the Sample
From February 26 onward, only symptomatic contacts of COVID-19 confirmed cases were tested with RT-PCR (44 RT-PRC tests were conducted before February 26; 1,947 afterwards).

RT-PCR	Serological assay (IgG)	Total
Performed	Not performed	1,364
Positive	-	632
Not performed	Performed	3,493
-	Positive	1,755
Performed	Performed	627
Positive	Negative	5
Negative	Positive	137
Positive	Positive	295
Negative	Negative	190

eAppendix 2. Statistical Analysis

Contact tracing data were combined with test and clinical outcomes of close contacts associated with each index case and used to estimate the risk of developing symptoms and critical disease by age after SARS-CoV-2 infection. We categorized contacts in five 20-years-age groups (0-19 years, 20-39 years, 40-59 years, 60-79 years, 80+ years). The likelihood of developing symptoms and critical disease after infection stratified by age and gender were defined as the proportion of symptomatic infected individuals and critical patients among the total number of infected individuals. Exact binomial test was applied to compute confidence intervals for different considered strata.

We considered an ensemble of nested logistic regression models where the clinical outcome of positive close contacts is considered as the response variable and possible covariates include:

1) the age group of the contact;
2) the gender of the contact;
3) a binary variable defining whether the index case in the cluster was symptomatic or not;
4) the number of symptomatic individuals in the cluster.

Specifically, to account for the clustering in the binary data, we applied generalized linear mixed-effects models (GLMM) with logit link specified as follows:

$$m(\mu_{ic}) = \alpha + \beta_1 A_{ci} + \beta_2 G_{ci} + \beta_3 S_{ci} + \beta_4 N_{ci} + u_c$$

where m is the logit link function, α is the intercept, A_{ic}, G_{ic}, S_{ci} and N_{ci} denote respectively the fixed effects of age group an individual i belongs to, the gender of the contact, a binary variable indicating whether the exposure took place in a cluster with a symptomatic index case, and the number of symptomatic infections in the cluster; u_c is the cluster-specific random effects and $\mu_{ic} = E(Y_{ci}|u_c)$ is the mean of the response variable Y_{ci} for a given value of the random effects. The model used to interpret the observed data was selected by adopting a stepwise procedure where the goodness of fit of two competing statistical models was assessed on the basis of likelihood ratio tests. The model selected to investigate the likelihood of developing symptoms after infection includes the age group and gender of the contact and the number of symptomatic individuals in the cluster. The model selected to investigate the likelihood of developing critical disease after infection includes the age group and gender of the contact. Risk ratios of experiencing symptoms and critical disease were computed given the covariates. Resulting means were compared by Tukey post-hoc test. The statistical analysis was performed with R (version 3.6.2).

Additional results and sensitivity analysis.

Results of the generalized linear mixed-effects model for the development of symptoms after SARS-CoV-2 infection are reported in eFigure 2A,B.
In our baseline analysis, contacts identified by more than one positive case were associated with the cluster of

© 2021 Poletti P et al. *JAMA Network Open.*

the first identified positive index case. The statistical analysis was replicated for 1,000 datasets where contacts with multiple index cases were randomly assigned to one of the clusters they belonged to. Estimates obtained in this sensitivity analysis were compared to those obtained in the baseline analysis in terms of mean and 95%CIs of estimated RRs of developing symptoms for different age groups (see eFigure 2C).

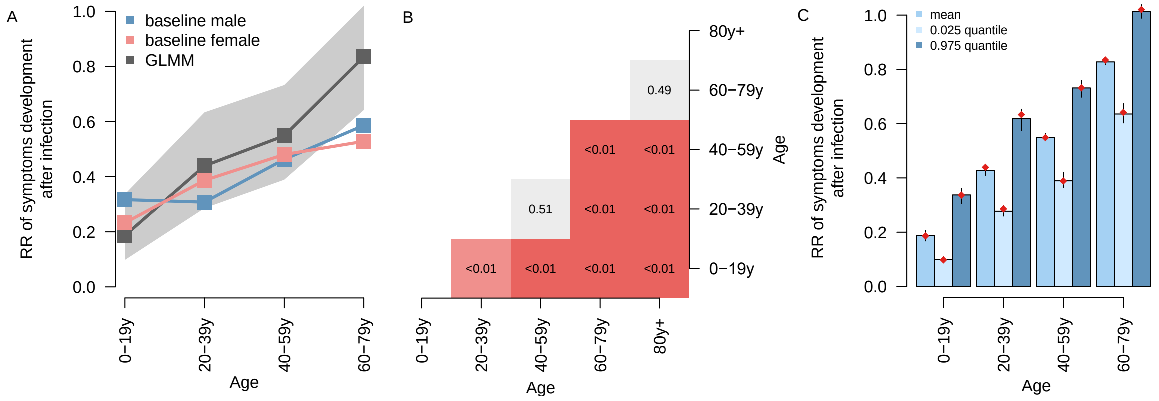

eFigure 2. Results of the Generalized Linear Mixed-Effects Model for the Development of Symptoms After SARS-CoV-2 Infection

A) Relative risks (RR) of developing symptoms by age group (with the last age group, 80+, as reference) estimated employing a generalized linear mixed-effects model (GLMM) with logit link (shaded area shows 95%CI). The RR increases with the number of symptomatic infections occurred in the cluster (p-value<0.01). **B)** Significance of different risk of developing symptoms across multiple age groups; the number displayed for any possible combination of two age groups i and j represents the p-value associated with the difference between group i and j as obtained by using on-way ANOVA, followed by post-hoc Tukey test. **C)** Estimates for the RRs of developing symptoms after infection across different age groups compared to participants older than 80 years, as obtained by randomly assigning these contacts to one of their index cases. Black lines show the entire range for mean estimates and 95%CIs obtained from the 1,000 datasets simulated in the sensitivity analysis; blue bars represent the average values over the 1,000 simulated datasets; red diamonds show the corresponding estimates obtained in our baseline analysis, where contacts with multiple index cases were assigned to the first identified positive index case.

eReferences

[1] World Health Organization. (2020). Contact tracing in the context of COVID-19: interim guidance, 10 May 2020. Available from: https://apps.who.int/iris/handle/10665/332049

[2] Corman VM, et al. (2020) Detection of 2019 novel coronavirus (2019-nCoV) by real-time RT-PCR. Euro Surveill 25(30): 2007303.

[3] Geurts van Kessel C, et al (2020). Towards the next phase: evaluation of serological assays for diagnostics and exposure assessment. medrxiv https://www.medrxiv.org/content/10.1101/2020.04.23.20077156v2.full.pdf

[4] Bonelli F, et al. (2020) Clinical and analytical performance of an automated serological test that identifies S1/S2 neutralizing IgG In Covid-19 patients semiquantitatively. Journal of Clinical Microbiology 01224-20. doi: 10.1128/JCM.01224-20

© 2021 Poletti P et al. *JAMA Network Open.*
